# Non-Compaction Cardiomyopathy and Multiple Sclerosis: Associated or Independent Diseases? A Case Report

**DOI:** 10.3389/fcvm.2022.871350

**Published:** 2022-05-06

**Authors:** Juan Manuel Muñoz Moreno, Carlos Holguin Palacios, Carlos Lobato Jeri, Sebastian Reyes Villanes, Wilson Peralta Ramos, Miguel Reyes Rocha

**Affiliations:** ^1^Department of Cardiology, Hospital Nacional Edgardo Rebagliati Martins, Lima, Peru; ^2^Department of Cardiology, Instituto Nacional Cardiovascular, Lima, Peru; ^3^Department of Neurology, Hospital Nacional Edgardo Rebagliati Martins, Lima, Peru

**Keywords:** heart failure, multiple sclerosis, myocardium, non-compaction cardiomyopathy, left ventricular dysfunction

## Abstract

Non-compaction cardiomyopathy (NCCM) is associated with neuromuscular disorders; however, there has been little investigation on its association with other neurological diseases, such as multiple sclerosis. We present the case of a 46-year-old woman with a history of multiple sclerosis who developed heart failure and was diagnosed with non-compaction cardiomyopathy.

## Introduction

Non-compaction cardiomyopathy (NCCM) is characterized by a bilayer myocardial structure, a thin compacted epicardial layer, and a thicker spongy endocardial layer with prominent trabeculations and deep intertrabecular recesses (1). There is an embryonic hypothesis that attributes the development of this pathology to arrested compaction of the loose meshwork of the myocardial primordium during embryonic development, causing the persistence of deep trabecular recesses in the myocardial wall (2, 3). However, there is not enough evidence to affirm this hypothesis, as argued by Jensen et al. (4), who found that adult hypertrabeculated left ventricles (LVs) were different from the embryo in that they were less trabeculated (15–40% vs. 55–80%) and had thicker trabeculae.

When NCCM is systematically investigated, neuromuscular disorders are found in 80% of cases, and associated mutated genes have also been described (5); unlike what happens with multiple sclerosis (MS), where the common pathogenesis is still unknown. To our knowledge, this is the second reported case of NCCM associated with MS (6).

## Case Description and Diagnostic Assessment

**Figure 1:**
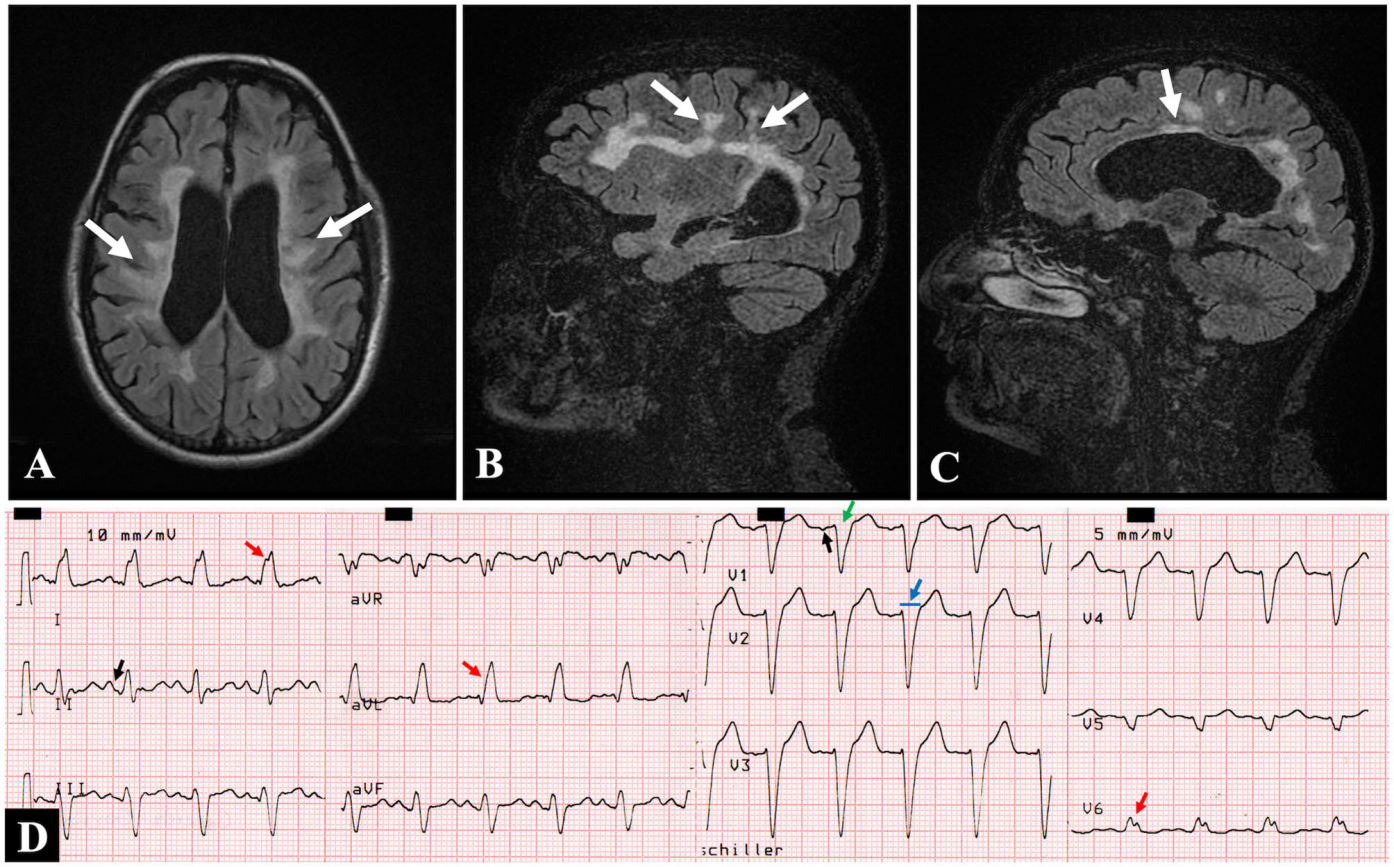
Brain magnetic resonance imaging. **(A)** Axial and **(B,C)** sagittal views in FLAIR sequence showing demyelinating lesions consistent with multiple sclerosis: perpendicular to the lateral ventricles **(A)**, periventricular **(B)**, and corpus callosum **(C)** (white arrows). **(D)** Twelve-lead electrocardiogram showing sinus tachycardia (125 beats/min), left atrial dilatation [P mitrale in II, and deepening of the terminal negative portion of the P wave in V1 (black arrows)] and left bundle branch block [QRS duration of >120 ms (blue arrow), lead V1 with a dominant deep S wave (green arrow); broad, notched R wave in V6 and I, and monophasic R wave in aVL (red arrows)]. ms, milliseconds.

A 46-year-old woman was admitted to our hospital for pelvic inflammatory disease complicated by pelvic peritonitis; she underwent exploratory laparotomy with right salpingectomy, and double antibiotic therapy was started. In the postoperative period, she presented with stage 2 acute kidney injury (creatinine: 2.45 mg/dl, urea: 74 mg/dl) and new-onset dyspnea. Her past medical history revealed a history of relapsing-remitting MS (Figures 1A–C) diagnosed 15 years ago and treated with azathioprine and prednisone. She had no previous history of hypertension, diabetes, or dyslipidemia. In the important family history, one of her sisters also has MS. She did not give detailed information about heart disease in her family. Physical examination showed spastic paraparetic gait with little steps, and auscultation found inspiratory crackles in the lower third of both lungs, rhythmic heart sounds with no murmurs or gallops. Blood pressure was 120/80 mmHg, pulse rate was 120 beats/min, respiratory rate was 16 breaths/min, and oxygen saturation was 95% with nasal cannula set at 2 L/min. The electrocardiogram (ECG) showed sinus tachycardia and complete left bundle branch block (LBBB) (Figure 1D).

The Pro-B-type natriuretic peptide plasma level was elevated (Pro-BNP: 1,038 pg/ml, normal < 125 pg/ml). High-sensitivity troponin T and dimer D concentrations were within the normal range. The complete blood count showed a decrease in white blood cell count (11.39 10^3^/ul; at the beginning 19.60 10^3^/ul), and the acute kidney injury had resolved (creatinine: 0.98 mg/dl; urea: 44.5 mg/dl).

In the first postoperative evaluation, we observed signs and symptoms of volume overload, sinus tachycardia, and LBBB on the ECG. In this setting, the initial differential diagnosis was directed toward acute decompensated heart failure (ADHF) secondary to cardiomyopathy. The history of MS raised the suspicion of an undetected underlying structural heart disease. On the other hand, other differential diagnoses were considered, such as atelectasis that was ruled out in the chest X-ray, vascular causes like pulmonary embolism since the D-dimer was in the normal range, and systemic causes like sepsis of abdominal origin, because of the absence of fever and a marked decrease in infectious markers with double antibiotic therapy.

**Figure 2:**
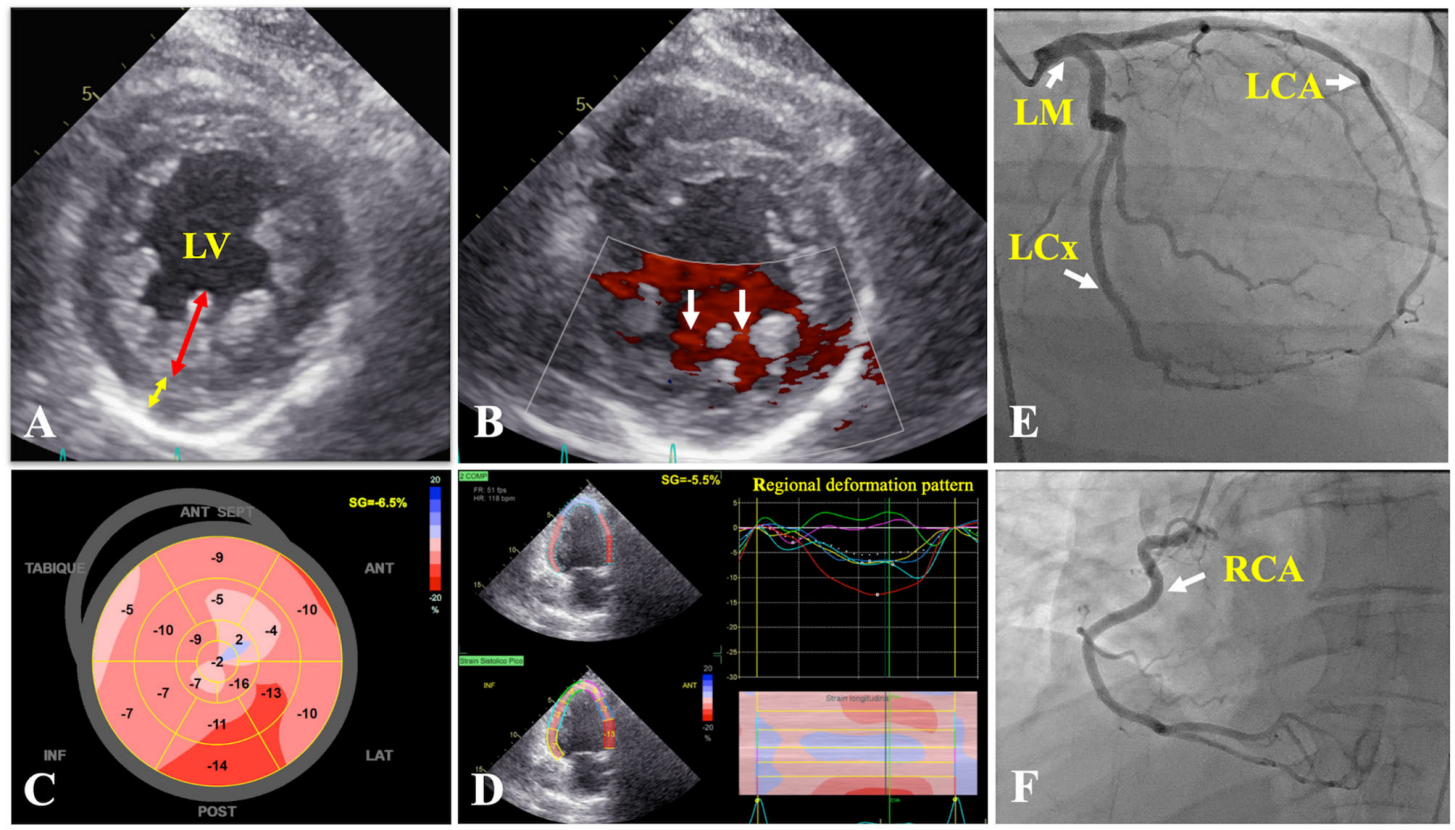
Transthoracic echocardiogram. **(A)** Parasternal short axis of the mid left ventricle demonstrating two-layer myocardial structure, with a ratio of ticker non-compacted (redline) to thin compacted (yellow line) myocardium >2 at end-systole in the inferolateral segment. **(B)** Flow between intertrabecular recesses by intraventricular blood on color Doppler (white arrows). **(C)** Reduced global longitudinal strain (−6.5%). **(D)** Two-chamber view showing a regional deformation pattern with a markedly decreased myocardial deformation in apical segments (green and purple curves) in comparison to basal segments (red and yellow curves). Coronary angiography: coronary arteries without lesions **(E,F)** (white arrows). LCA, left coronary artery; LCx, left circumflex coronary artery; LM, left main coronary artery; LV, left ventricle; RCA, right coronary artery.

Transthoracic echocardiogram (TTE) demonstrated a left ventricular ejection fraction (LVEF) of 30%, mildly dilated, diffusely hypokinetic, prominent trabeculations of LV, and a non-compacted to compacted (NC/C) myocardium ratio of 2.75 in end-systole at inferolateral mid-ventricular level (Figures 2A–D), so the diagnosis of NCCM was realized. On the sixth hospital day, coronary angiography revealed that coronary arteries were normal (Figures 2E,F). Right-sided cardiac catheterization showed normal cardiac index and absence of pulmonary hypertension.

The clinical status was stabilized with diuretic therapy, enalapril 5 mg bid, spironolactone 25 mg OD, and bisoprolol 5 mg OD for heart failure, with reduced ejection fraction (HFrEF) were progressively initiated. During hospitalization, she was evaluated by neurology to regulate her treatment for MS and avoid any relapse crisis, which did not appear. The evolution of the patient was favorable, and she was discharged on the 14th day of hospitalization.

**Figure 3:**
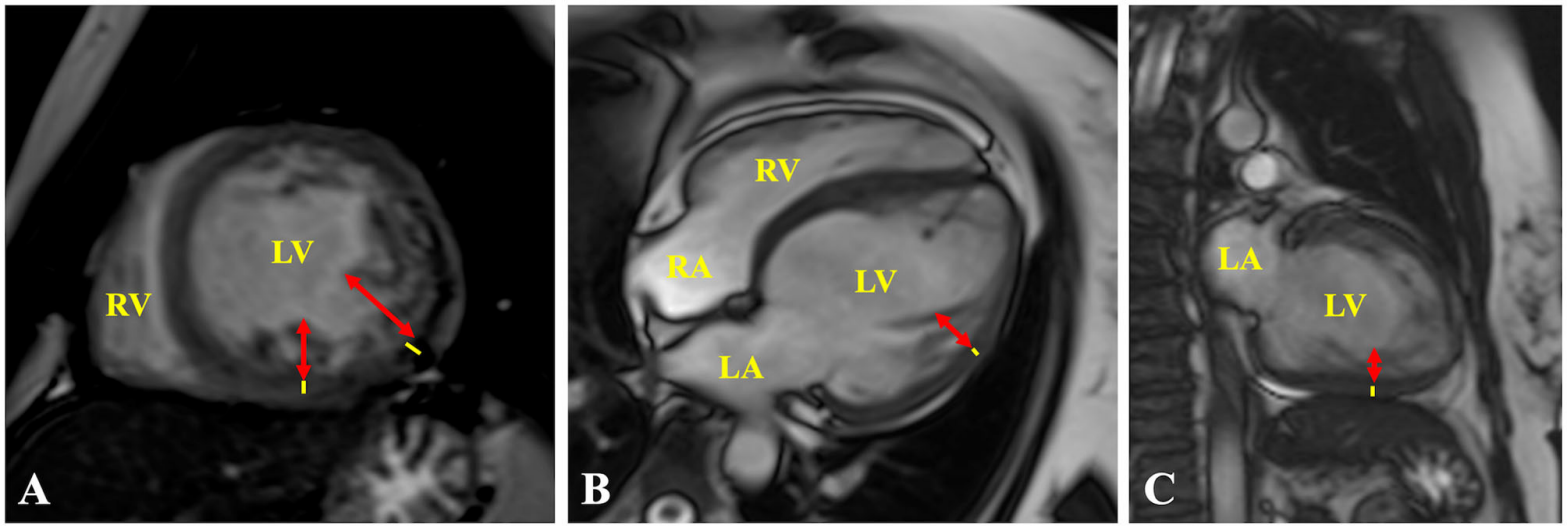
Cardiac MRI. **(A)** Short axis, **(B)** 4-chamber, and **(C)** 2-chamber views showing representative measurements of the ratio of ticker non-compacted (redlines) to thin compacted (yellow lines) myocardium >2.3 at end-diastole, in the **(A)** mid-inferolateral, **(B)** mid-anterolateral, and **(A,C)** mid-inferior segments. LA, left atrium; LV, left ventricle; RA, right atrium; RV, right ventricle.

**Figure 4:**
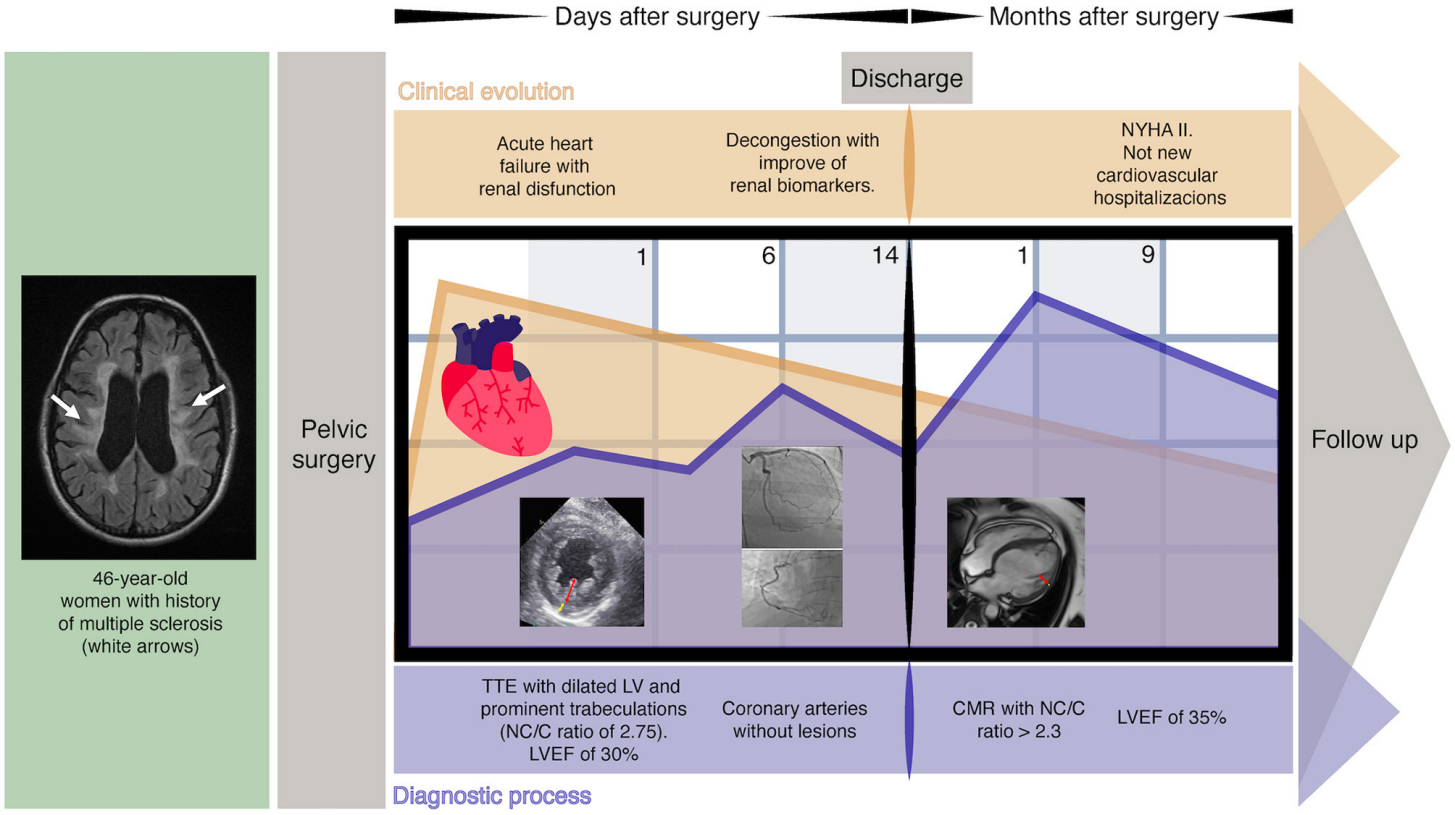
The timeline of events. CMR, cardiac magnetic resonance; LV, left ventricle; LVEF, left ventricular ejection fraction; NC/C, non-compacted to compacted; NYHA, New York Heart Association; TTE, transthoracic echocardiogram.

Cardiovascular magnetic resonance (CMR) imaging was performed 1 month after discharge to confirm the diagnosis and demonstrated positive diagnostic criteria for NCCM; additionally, the study did not show late gadolinium enhancement (LGE) (Figure 3). Currently, after 9 months of outpatient follow-up, the patient remains in optimal medical therapy. Bisoprolol was titrated to 5 mg bid, and dapagliflozin 10 mg OD was started. The LVEF improved slightly to ~35%, in NYHA class II, and she did not require hospitalizations for ADHF. A timeline is showcased in Figure 4.

## Discussion

Non-compaction cardiomyopathy (NCCM) is classified as primary genetic cardiomyopathy by the American Heart Association (AHA) and unclassified cardiomyopathy by the European Society of Cardiology (ESC), with an adult prevalence of 0.014% (2). NCCM is associated with neuromuscular disorders; however, there has been little investigation on its association with other neurological diseases, such as MS. The first case report of NCCM and MS was published in 2009 (6). However, it should be noted that we are proposing for the first time a possible association pathway, which is through tenascin C, on which some research has already been conducted in NCCM and MS independently for now. This is a glycoprotein of the extracellular matrix that is temporarily expressed in the heart during embryonic development and regulates several cellular functions, including embryogenesis and nerve regeneration (7). In adults, this protein is reexpressed under pathological conditions regulated by inflammation, such as NCCM and MS, and is found in elevated serum levels (7, 8).

Momčilović et al. (9), in an experimental MS model with mice, evidenced that tenascin C is involved in the pathogenesis of central nervous system autoimmunity, through Th1 and Th17 cells.

The NCCM can be grouped into seven phenotypical subtypes (3), among which our case is associated with LV dilation and dysfunction at baseline. The interaction between MS relapses and myocardial stunning through a sympathetic outburst has been proposed, similar to what occurs in stress cardiomyopathy, where it has been found in common that demyelinating lesions compromise the medulla oblongata that controls the neurovegetative cardiovascular center (10). Unlike this, in our patient, the demyelinating lesions found (Figures 1A–C) did not affect the brainstem that includes the medulla oblongata; therefore, we rule out this alternative hypothesis to explain the reduced LVEF in our case, and we suggest that this is probably due to the advancement of the NCCM itself.

The TTE fulfills a fundamental value in the initial diagnostic approach, being Jenni's criteria the most widely used, and evaluates a two-layer ventricular myocardium with thick non-compacted and thinner compacted myocardium, generating an NC/C ratio >2 in end-systole on short-axis parasternal view (11). These alterations affect predominantly the mid-lateral, mid-inferior, mid-anterior, and apical LV areas (12). Finally, prominent excessive trabeculations and the flow between the deep intertrabecular recesses by color Doppler and excluded coexisting structural cardiac abnormalities are also considered (11). Cardiac magnetic resonance (CMR) allows us to confirm the diagnosis with 86% sensitivity and 99% specificity, following Petersen's criteria that consider an NC/C ratio >2.3 in end-diastole (11). Both criteria were met by our patient. The differential diagnosis by TTE was made with a normal variation of myocardial trabeculations, defined as less than 3 trabeculations located in the LV apex, also with LV apical thrombi, false tendons, aberrant chords, cardiac tumors, hypertrophic cardiomyopathy (HCM), and dilated cardiomyopathy (DCM) (12, 13). The LV apical thrombi are distinguished by their higher echogenicity compared to myocardium; false tendons and aberrant cords usually cross the LV cavity (13). None of these findings were observed in our patient, neither was any cardiac tumor. Recently, the characteristics of Speckle Tracking in NCCM began to be studied, evidencing a reduced global longitudinal strain, with greater involvement of the apical segments, generating a significant basal-to-apical gradient, useful to differentiate it from HCM and DCM (11, 13). Additionally, it differs from HCM, since trabeculations and crypts that can mimic NCCM are mainly limited to the basal ventricular septum or the posterior wall (13).

The ECG findings are non-specific and include left ventricular hypertrophy, inverted T waves, and different types of bundle branch blocks (2, 12). Currently, there is no specific therapy for patients with NCCM and HFrEF, so it is recommended to follow the heart failure (HF) management guidelines (12). Consequently, our patient received optimal medical therapy. According to the 2021 European HF guidelines, an implantable cardioverter-defibrillator (ICD) should be considered to reduce the risk of sudden death and all-cause mortality in patients with symptomatic HF (NYHA class II–III) of non-ischemic etiology and LVEF ≤ 35 % after at least 3 months of optimal medical therapy (OMT), and with a life expectancy greater than 1 year with good functional status (class IIa, level of evidence A). If a patient is scheduled to receive an ICD and is in sinus rhythm, with an LBBB, cardiac resynchronization therapy with defibrillator (CRT-D) is recommended if the QRS is ≥ 150 ms to improve symptoms and reduce morbidity and mortality (class I, level of evidence A) (14). In our case, we are still titrating OMT; and for ICD implantation, a minimum of 3 months of OMT is recommended to assess whether the LVEF fails to increase to >35%, so that in the following controls, according to the evolution of the patient she will be reassessed for the need to implant an ICD and/or CRT-D.

Anticoagulation is mandatory in all patients with NCCM with atrial fibrillation, previous thromboembolic events, or LV thrombus (class I, level of evidence B), whereas in those with NCCM and only LV dysfunction, it may be considered (class IIb, level of evidence B) (1, 15). Patients with NCCM and LV dysfunction, presenting with LGE on CMR imaging, compared to those without LGE, experienced stroke more frequently (1). Fortunately, in our case, the presence of LGE was not observed. Starting with direct oral anticoagulants was proposed to the patient and, as a second option, a vitamin K antagonist. However, both options were rejected because of economic issues and the risk of bleeding, respectively.

The prognosis depends on the development of HF or the need for heart transplantation; likewise, there is a variety of predictors associated with poor outcomes that include the presence of advanced age, inpatient's NCCM diagnosis, NYHA functional class III–IV, and LVEF <31% (12, 16). Vaidya et al. (17) found that age, LVEF <50%, and non-compaction extending from the apex to the mid or basal segments were associated with all-cause mortality.

It was suggested that first-degree relatives undergo echocardiographic screening; however, because of health insurance issues and living in another locality in Peru, this has not yet been possible. When we were evaluating the family history, the patient mentioned that one of her sisters also had a diagnosis of MS and was being evaluated in a private clinic.

## limitations and Strengths

### Strengths

This case serves to propose a possible pathway of association between NCCM and MS, through Tenascin C. In addition, it highlights the importance of performing a cardiovascular evaluation that includes echocardiographic screening in all patients with MS to timely identify NCCM, and particularly the dilated LV phenotype with reduced LVEF, to initiate OMT and prevent HF progression.

### Weaknesses

Because of the lack of advanced genetic studies, it was not possible to perform genetic screening on our patient. Tenascin C was not measured in our patient because of the lack of resources in our reality. It is necessary to carry out studies on large population groups to confirm this possible association between the pathologies described.

## Future Directions

The NCCM is possibly associated with MS; however, the exact pathophysiological mechanisms that explain the association are not clear, and with the present case, we would like to encourage the research for evidence through studies with large population groups on Tenascin C.

## Conclusion

Patients with NCCM with a phenotype of dilated LV with reduced LVEF must be diagnosed promptly to initiate OMT and avoid the progression of HF.

## Data Availability Statement

The original contributions presented in the study are included in the article/supplementary material, further inquiries can be directed to the corresponding author.

## Ethics Statement

The studies involving human participants were reviewed and approved by Hospital Nacional Edgardo Rebagliati Martins. The patients/participants provided their written informed consent to participate in this study.

## Author Contributions

JM contributed to conception and design of the article. JM and CH wrote the first draft of the manuscript. JM, CH, and CL wrote sections of the manuscript. All authors contributed to manuscript revision, read, and approved the submitted version.

## Conflict of Interest

The authors declare that the research was conducted in the absence of any commercial or financial relationships that could be construed as a potential conflict of interest.

## Publisher's Note

All claims expressed in this article are solely those of the authors and do not necessarily represent those of their affiliated organizations, or those of the publisher, the editors and the reviewers. Any product that may be evaluated in this article, or claim that may be made by its manufacturer, is not guaranteed or endorsed by the publisher.

## Abbreviations

ADHF: acute decompensated heart failure
CMR: cardiac magnetic resonance
CRT-D: cardiac resynchronization therapy with defibrillator
DCM: dilated cardiomyopathy
ECG: electrocardiogram
HCM: hypertrophic cardiomyopathy
HF: heart failure
HFrEF: heart failure with reduced ejection fraction
ICD: implantable cardioverter-defibrillator
LBBB: left bundle branch block
LGE: late gadolinium enhancement
LV: left ventricle
LVEF: left ventricular ejection fraction
MS: multiple sclerosis
NCCM: non-compaction cardiomyopathy
NC/C: non-compacted to compacted
NYHA: New York Heart Association
OMT: optimal medical therapy
TTE: transthoracic echocardiogram.
